# Abstract of the Oration in Surgery1Delivered at the Centennial Meeting of the New York State Medical Society at Albany, January 31, 1906.

**Published:** 1906-02

**Authors:** Roswell Park

**Affiliations:** Professor of Surgery. Medical Department, University of Buffalo


### SPECIAL ARTICLE.

### Abstract of the Oration in Surgery[1]

By ROSWELL PARK, A. M., M. D., LL. D.,
Professor of Surgery. Medical Department, University of Buffalo.

FEW anniversary celebrations have ever been held in this country that have had or can have a wider significance than that in which we are now participating. It marks not only the centennial of a great and influential Society, in the greatest and most influential State in our Union, but it signalizes also the reunion of men and professional interests which have been too long kept apart by false sentiment and inability to maintain a common point of view. The occasion, then, is so memorable that I feel almost aghast at the task assigned me of trying suitably to characterise the surgical work which has been accomplished, especially within our own limits within the past century, at the same time, I value most highly the compliment conveyed by the invitation to deliver the centennial oration. Growth in population in this State has almost kept pace with advance in the natural sciences. When Dr. Fisher delivered his Presidential and anniversary address before this Society in 1875, he reminded us that when the law which created it was enacted, the State of New York was then divided into thirty-five counties instead of into sixty, as at in the State, such as they were, not a few of whom "sadly needed told also that there were then about eight hundred members within the State such as they were, not a few of whom "sadly needed regulating." Buffalo, the Western metropolis of the State, was then a village of 1500 souls, whereas it now comprises over one-third of a million of inhabitants. This was, to be sure, before the second war with Great Britain, in the course of which our Western frontier became a line of no small strategic importance.

1. Delivered at the Centennial Meeting of the New York State Medical Society at Albany, January 31, 1906.

In 1820, Sydney Smith voiced that memorable cynical and sceptical query of his as to what Americans had done, not only asking "Who reads an American book," but saying "What does the world yet owe to American physicians and surgeons? What new substances have their chemists discovered?" It must be said that at that time they had done little, and assuredly that little had not come to Smith's knowledge, partly because of his own prejudice, and partly because information traveled slowly in those days. But if the great literateur and cynic of his day could come to life again, and familiarise himself with what Americans have done since he disappeared from the world's stage, he would have to express himself in very different terms. In reply to his query as to what our chemists have discovered we could point, for instance, to chloroform, which was discovered by Guthrie, of Sackett's Harbor, and to numerous other anaesthetics which, if not first discovered in this country, were first utilised here. I think we may all well agree with Dennis when he says: "It is an actual fact that if you were to strike from the notable surgical achievements and writings of the world what has been contributed by America during the past few decades, there would be left but little new and original work for the older nations to claim as their own."

I do not know how I may better characterise one aspect of modern surgical progress than by quoting from an address delivered at the opening of The University College Hospital, in London, in the fall of 1873, by Mr. Erichsen, at that time justly considered to be one of the foremost of living British surgeons. In this address he dealt largely with the subject of *Finality in Surgery,* and expressed himself regarding it as follows: (London Lancet, October 4th, 1873, pp. 489).

In no department of science had intellectual development brought greater changes in a comparatively brief space of time than in medicine. In surgery it appeared to him that there were two great schools :The Practical and The Scientific. A generation back The Anatomical School had reached its acme. Thirty-five years previously, surgery, as a manipulative art, had fallen into a sluggish and inactive state, and no advances had been made in the two greatest operations of surgery since Cheselden, a century before, had introduced his operation, or since Hunter had immortalised his name by his procedure for aneurism. He then went on to say:: "But there must be a final limit to development of manipulative surgery. The knife can not always have fresh fields for conquest and, although methods of practice may be modified or varied, and even improved to some extent, it must be within a certain limit. *That this limit has nearly, if not quite,*

*been reached will appear evident if we reflect upon the great achievements of modern operative surgery.* Very little remains for the boldest to devise or the most dexterous to perform." After which he very briefly alludes to the studies of Lister, who at that time was pursuing his painstaking researches almost within gun-shot distance of the place where Erichsen stood while making these remarks, researches which revolutionised the surgery of his day, and made that not merely possible but easily practicable which before had seemed unjustifiable or even criminal.

In the marvellous surgical accomplishments of the past thirty years we see the tremendous effect of the development of a theory and its application, since not until the *germ theory of disease* had been tested thoroughly and proven reliable by scientists, (among whose names that of Pasteur must be always mentioned first), could rational methods be adopted for combatting the minute and deadly enemies by which mankind is always surrounded.

Another theory, which must be credited to the past century has had much to do with explaining many problems that interest us, perhaps not so much as surgeons, but as scientists—namely, *the theory of evolution.* In those earlier days this theory was rarely correctly stated; too often at present it is not correctly judged, and comparatively few can grasp its entire significance. In its briefest expression it means the establishment of an organic continuity between the lowest and the highest forms of life; but, taken in conjunction with an accurate knowledge of embryology, it has much to do with the explanation of many of the congenital defects and arrests of development with which we as surgeons have to deal.

The two greatest discoveries of the past century are one of British, the other of American origin; both, in other words, of Anglo-Saxon offspring. I allude, of course, to Anesthesia and Antisepsis. While New York State had no conspicuous part in the early development of either, it must nevertheless be said that New York surgeons have been quick and prompt to seize upon the discoveries thus placed before them, and have been enthusiastic in their later development and improvement. When this Society was organised it was as necessary then as ever to intoxicate or narcotise patients before operations, and still to see them suffer the agonies of the torture chamber, agonies which no amount of sympathy or skill of the day could mitigate beyond a certain point.

When Wells introduced nitrous oxide gas, when Morton promoted the use of ether, and when Simpson first administered chloroform, there were placed before the profession, within a short period of time, three of the greatest blessings which a kind Providence has ever vouchsafed to mankind. It is late in the years now to describe the horrors of the old time operating room, or even to quote the vivid descriptions either of surgical clinics of the time or of the feelings of the operators who took part in them. If one desires to seek descriptions of such gruesome scenes he may find them, but will read them as one reads accounts of the Inquisition and its tortures. But this has all disappeared, and that the performance of an operation can now be robbed of all that makes it unpleasant and disquieting is one of the marvels of the century and one of its greatest accomplishments.

Indeed it means much more than the abolition of suffering; it means that that which was previously beyond human endurance is now placed easily within it; it means that that which was formerly considered inhuman and unjustifiable is now regarded as humane and scientific. But to this audience and at this time it is unnecessary to enlarge further upon this aspect of the century's achievments.

The introduction of anesthesia took away the most imperative need for rapidity, and the introduction of antisepsis required more time for attention to detail. In consequence, there grew up a certain school of men who seemed to feel that time was no object in operating, and that so long as the patients were comfortably asleep the principal indication was thereby met. It did not take long, however, for the profession to learn that the anesthetic itself is by no means a harmless agent, to be administered for indefinately continued periods, but that it has its own disadvantages, and that the shorter the time during which the patient is kept under it the better for him. We have accordingly reverted somewhat to earlier conditions, and have learned that while time should not be sacrificed to other conditions of greater importance it nevertheless is of value, and that he is the best operator who, other things being equal, saves time so far as he can.

But what shall now be said of the changes effected by the introduction of antiseptic methods? There are those who can look back further than I can, and yet it has happened that quite within my easy recollection the whole aspect of surgery has been changed by the work of Lister and his co-workers. I can remember my first season as house surgeon, in one of the largest and newest of the Western hospitals, when throughout that long and, to me, sad season, scarcely one patient was submitted to any major or semi-serious operation who did not die of blood poisoning. I saw men die after what seemed to me even minor operations. Scarcely a patient entered the hospital with a compound fracture whose doom was not sealed. Tracheotomy was a useless performance; trephining was interesting as a spectacle, but useless as a resource. Even internal urethrotomy became a fatal procedure; and so throughout the list. This was in the years of grace 1876 and 1877.

What, then, can be said of the men and the method by whom and by which all of this horrid reality was changed, so that the practice of surgery became a pleasure rather than a solemn duty. The younger men who have entered the profession within recent years can never appreciate what it meant to practise in those earlier days, which yet are not so long past. Only he who has seen case after case of compound fracture go to the ground, as the result of preventable infection, can fully appreciate the neccessity for the precautions which we take today, or can have within him that true feeling of reverance and love for the man who brought about this revolution. That to Pasteur a monument has been erected, and that Lister has been made an English lord, constitute but a small part of the respect and honor we should yield them. This, perhaps, is the best which a government of the aristocracy can do, but there is a higher position and a nobler one which they occupy, and ever will, in the heart of every surgeon, as of every one who is capable of appreciating what has been accomplished through their life and labors.

What shall be said next of the extension of research by which surgical pathology has been wedded to the study of surgery as an art, and made an inseparable part of a surgeon's self preparation for his work? Next to asepsis and anesthesia the evolution of the surgical laboratory, and the work done therein, constitute the most conspicuous advance in the science of surgery made during the past century. Not that surgical pathology is by itself a subject distinct from general pathology, but that it implies the application of the principles of general pathology to surgical problems. Here, as in many other places. the microscope and the test tube for minute work, and the introduction of experimental methods, both in the study of disease and the perfection of technique, have afforded opportunity for most wonderful discoveries and most striking progress.

I would divide the work done within a surgical laboratory under two headings,—the minute and the gross.

Under the title of *Minute Work* I would include that which is purely *histological,* that which woul be ranked as *bacteriological,* and that which may be perhaps best spoken of as *biochemical.* By the Histological, the nature of the tuberculous, leprous, actinomycotic and numerous other morbid processes has been singularly cleared up. The works that have been published, for instance, on tumors alone, within the past four decades, would fill more than one shelf in any large library.

Under the heading Bacteriological work, I would include the researches which resulted in that most important advance which we sum up under the title The Theory of Sepsis and the Practice of Asepsis. The work done by Nicolaier and Kitasato on the origin and nature of tetanus, by Villemin and Koch in the study of tuberculosis, and by Neisser on the specific nature of the gonococcus, also come under this heading.

Of the Biochemical researches which concern the surgeon, we cannot speak without thinking of Tuberculin, which proved in the main a disappointment, and yet which had its important role to play, and of the antitoxins with which we now treat diphtheria, septicemia and tetanus, as well as the serums or similar preparations like those, for instance, devised by Calmette and Vital respectively in their so-called 'Antivenene' and 'antiophidic serum.' These serums appeal to us perhaps but slightly or not at all, yet think what they mean in India, where more than 20,000 people perish every year from the bites of venomous serpents. Serotherapy, then, has come to stay, as one of the most conspicuous illustrations of the results worked out entirely within the walls of surgical laboratories, the product alike of inductive reasoning and experimental research.

Of the gross work of a surgical laboratory I can give only illustrative examples. When Senn began his work and published his first experiences with intestinal anastomosis he employed methods which were crude, compared with those now in vogue, but they have since been widely extended and made available in various ways, of which in the beginning no one dreamed. The anastomotic method has been extended not merely from one part of the intestine to another, but from gall-bladder to the bowel,—though this is today rarely done,—and from the stomach to stomach, to the duodenum, or to any other convenient part of the alimentary canal, while even by transdiaphragmatic methods it has been suggested to anastomose the stomach to the oesophagus above the diaphragm.

The lessons thus taught by this work upon the bowel led to more vigorous prosecution of experimental work upon diverting other currents or other functions of the body and rearranging them to suit new needs. Thus, for instance, the method of nerve grafting has been widely extended, and made available in a large number of cases of infantile and other palsy, as well as for establishing nerve communication around injured or exsected nerves. Of this perhaps there is no more conspicuous illustration than the treatment of facial palsy by transplantation of the hypoglossal or the spinal accessory into the facial nerve. Nerve suture also has become a general expedient and one which it is criminal to neglect when demanded.

Similarly, tendons have been disengaged from their proper insertions and replaced anew, so as to utilise the power of muscles still active, and atone for paralysis of parallel or even antagonistic groups, Tendon suture and tendon grafting, then, have become every day expedients for the correction of deformity and restoration of function, in which they have been wonderfully successful.

We have learned that among the tubular connections of the body, the ureters,. like the intestines, may be resected, transplanted or anastomosed, as occasion may demand.

Similarly, even the blood vessels have not escaped the investigator's attack, and we have found that they may be sutured, either longitudinally or even by end to end suture, as the researches, especially of Murphy, brilliantly have demonstrated.

No more remarkable illustrations of what can be accomplished, at least in the laboratory, have been reported than those from the Hull Physiological Laboratory of Chicago University, by Carrel and Guthrie (*Am. Medicine,* Dec. 30, 1905 pp. 1101). These experiments include such feats as reversal of circulation in veins and arteries, replantation of completely severed limbs and transplantation of the kidney and thyroid. Such experiments as the following, for instance, were performed: The heart of a small dog was extirpated and transplanted into the neck of a larger one, by anatomosing the cut ends of the jugular vein and of the cartoid artery to the large vessels of the heart. The circulation was re-established through the heart, and it showed fibillary contractions, beating at the rate of 88, while the normal heart was beating 100 per minute. Similarly a kidney was extirpated and transplanted into the neck, anastomosing the renal artery to the cartoid artery, the renal vein to the jugular, and causing the ureter to empty into the oesophagus. On the third day the circulation was normal and good excretion of urine was going on. So, too, with the thyroid, which was extirpated and replanted with reversal of the circulation. Forty days later it was easy to recognise systolic expansion of the gland.

Concerning blood pressure, on which none have done more brilliant work than Crile and Cushing of our own country, they have revolutionised our notions regarding surgical shock, they have shown the importance of both recording and maintaining blood pressure, they have demonstrated the value of the proper use of salt solution, and particularly they have given experimental proof of the marvellous nature of that singular substance, adrenalin.

Blood analysis has reached a degree of development and accuracy which cause it to rank with urinalysis, both in complexity and value. About all that was ever done in this direction one hundred years ago was an inspection of the so-called "buffy-coat" after venesection.

It is quite unnecessary to allude to the minute developments in urinalysis, which have not merely materially complicated it, but have made it a most valuable aid in diagnosis. There are those who claim to gather much of diagnostic value from a minute microchemical examination of the saliva, while the importance attaching to an examination of the cerebrospinal fluid is year by year increasing.

Almost our entire knowledge of cerebral localisation has been gained in the anatomical and surgical laboratory, by a score of diligent investigators, a list of whose names should, by common consent, be headed by Sir Victor Horsley. A great deal has been done in explanation of the various traumatic disturbances of the brain, included under the terms "contusion" and "compression." Perhaps in this line of investigation the French surgeons have taken the lead, and yet much has been done at home. So with regard to infectious processes within the cranium. The researches and publications of Sir William Macewen have brought us a clear knowledge of the subject which no one had attained until he began his work. The relation between the various sinuses of the face and cranium and the interior of the brain box have been thus impressed upon our knowledge, and in such a way that surgery may now usually anticipate the evils of intracranial suppuration, if only the surgeon be given an early enough opportunity.

In the surgery of the spinal cord it remained for American surgeons,—Harte of Philadelphia and McCosh of New York—,to show that suture of a completely divided spinal cord may be successfully made, with at least partial restoration of function, a demonstration which the ordinary laboratory has not yet afforded. Compression paralysis, moreover, has been made amenable to surgery, at least in many instances, whether a result of a morbid process, as in kyphosis, or of recent traumatism, and relief has been afforded which could not possibly have come in any other way.

Study next what has been done with special organs, and take first the heart. Modern surgery of the heart includes, not merely aspiration or opening and drainage of the pericardial sac, or incision into it for the purpose of breaking up adhesion, but includes actual attack upon the heart itself, there being now on record not a small number of instances of gun-shot or stab wounds where the heart has been exposed, the injury uncovered, and the heart substance sutured while still beating. A number of lives have been saved in this way which else would have been inevitably lost. More than this, massage of the organ has been successfully practised for collapse following anesthesia, and it has been shown to be not only feasible, but justifiable in such instances to open the thorax or the upper abdomen, if they have not already been opened, and, directly or through the diaphgragm, deliberately seize or manipulate the heart, combining such massage with artificial respiration, and compelling it to expel the blood which may empty into it; thus in several instances, snatching the patient from a yawning grave. Crile especially has shown how successful this measure is upon animals and has given the principle impetus to its practice in the operating room. The lungs have been made almost equally amenable to surgery. The earliest suggestions in this direction were made by one of our countrymen, Warren Stone. They were later improved upon by Estlander, who usually gets all the credit for it. Beyond this the lung is often invaded for abscess, gangrene, less often for hydatid cyst, tuberculosis or other granulomata, and even the more distinct tumors.

The operation of opening the larynx for suffocative conditions is an old one, but the extension of the method for any other condition than that threatening immediate death is a very modern development of the old idea.

On the oesophagus extensive operations have been successfully practised, and foreign as well as American surgeons, Bryant particularly, have shown the possibility of attack both upon the oesophagus and the lower end of the trachea; even the great bronchi; exposing them posteriorly through an opening made at the back of the chest.

Of the alimentary canal, what might not be said? We have learned that there is practically nothing which cannot be done with the alimentary tube from one end to the other, that no part nor section of it is essential for vital purposes, that the entire stomach may be removed, and that several feet of intestine can be easily and comfortably spared.

In addition to this, the past twenty-five years have brought us a revelation regarding the possible morbid activities of the vermiform appendix and the disastrous consequences which may ensue upon involvement of this little tube. For widespread gangrene of the neighboring intestine, I have myself in one instance removed nearly nine feet of bowel, in another six, and in two others but little less; in all four instances successfully, and I have further shown, after a study of reported cases, that it would appear that the more extensive intestinal resections yield an even larger amount of success than do the lesser.

Regarding the pancreas, a large amount of experimental work has been done, by which many problems have been cleared up, even if not finally solved. In this experimental work many Americans have been engaged, none among them, however, doing better or more telling work than my colleague Dr. Williams, in the University of Buffalo. The acute pancreatitis which might have been suspected twenty-five years ago on a *priori* grounds is now a demonstrated fact, with an equally demonstrated remedy.

Likewise the liver is no longer sacred ground, but in itself and in its adnexia is now frequently exposed to surgical attack, and with a marvelous amount of resulting benefit. Of the viscus itself it may be said that we not only open abscesses and hydatid cysts, but that we remove more or less extensive portions when involved in localised tumors or malignant growths. I have under my observation a patient from whom, nearly five years ago, I took away not only a large cancerous gall-bladder with enormous calculi, but a goodly portion of the surrounding liver. She is today doing her own housework and in all these respects as well as ever in her existence. When the liver is displaced it may be fastened in place, just as is done with the kidney.

The spleen becomes a legitimate object of surgery when crushed or ruptured by injury, when displaced so as to become troublesome, or when the site of abscess or of tumor. Moreover, its complete removal is indicated in cases of so-called splenic anemia, or Banti's disease, where the measure has given good results.

Very much has been done with the so-called ductless glands, among which the thyroid is the most prominent and most often attacked. All this work was at least begun and long continued in the laboratory, and not tested upon the human being until ample reason had been afforded by results of the experimental investigators. In consequence, the indications for operations upon the thyroid in the various forms of goitre are clearly defined, the only possible exception to this statement being in the case of exophthalmic goitre, where men still differ somewhat according to their notions concerning its pathology.

A large amount of laboratory and experimental work has been done with bones and joints. Murphy has accomplished much more of late by utilisation of muscles and fasiæ than anyone ever could expect to with ivory balls and sockets driven into bone ends.

There is another catagory of surgical procedures of which it might be said that they are the outcome rather of clinical observations than of laboratory work. Thus, for instance, in 1883, Van de Warker, of Syracuse, reported the case of an abdomen which he had opened, supposing that he had to deal with a minor form of hydrops. After incision and inspection it was discovered to be a case of tuberculosis of the peritoneum. The complete recovery of the patient led to the introduction of celiotomy as a curative measure, and the amount of good that it has accomplished is very great. Some surgeons simply open and evacuate, some wash out with salt solution, while, in my own experience, I have had the most encouraging results from complete evacuation and lavage and then the introduction of a credé lactate of silver solution in the abdominal cavity while closing it.

Operations for the radical cure of hernia have been practised from the earliest days of surgery, becoming perhaps a little less crude with each advancing century or surgical cycle. While every one desires to close the inguinal canal, there are so many ways of accomplishing this that one may well hesitate as to which is the best. Of late I have used, with gratifying success, when the length of the hernial sac permitted it, after ligation and fixation of its neck, either the entire sac, twisted into a small cord and used as a suture, being passed from one side of the ring to the other by means of a large needle with a large eye; or, when the sac is too large, I have not hesitated to make a strip from a portion of its structure and used this for the same purpose, after extirpating the balance. In this way I have found suture material of ample length of fresh animal tissue, which is sure to be aseptic, which utilizes something that else would otherwise be completely removed, and saves taking away a strip from the edge of the canal and thus widening or in any way weakening it.

Of laparatomy as a general measure we have lost all our dread. As performed for the relief of intussusception it was first done upon a negro slave by Wilson, in 1831. His patient had suffered for seventeen days; nevertheless the entangled intestine was succesfully released and the patient made a complete recovery (Dennis). It was not, however, until after Lister's triumph that abdominal section became by any means a safe operation. At present it is everywhere performed, unfortunately, too often by those who have had insufficient experience. If in any way the mortality at present attaching to it should seem unduly large, although in reality small, it is partly ascribable to the fact that too many of the younger surgeons of the country think to win their spurs in the profession by a series of cases of abdominal section, for which it may be that experience and training have not yet fitted them. The larger one's experience becomes in this direction the less certain he is as to the interior conditions from exterior examination, and the more guarded he becomes in the descriptions of what he expects to encounter. Thus it too often happens that a case which appears simple is complicated in a way to demand the ripest experience of an expert operator. The last quarter of the century has seen an extraordinary increase in the number of abdominal sections done in all parts of the world, for which there is perhaps much good reason. As the factor for safety has increased, one sentimental objection to its performance has disappeared, while the great advantage accruing by substitution of accurate knowledge acquired by the operative method for previous guesswork is obvious, even to many patients. Moreover the importance of early attack upon cases of cancer is more and more widely appreciated, and I fear no opposition to a statement, which may be regarded almost as a challenge, and one that I have frequently made, that a well grounded suspicion of intra-abdominal cancer justifies a well performed exploratory operation, directed, first, to its determination, and, second, if possible, to its removal. In cases of cancer of the stomach, for instance, to wait until diagnosis can be made by the sense of touch, from the outside, is to wait until the patient has passed the period of safety, whereas if, so soon as cancer is suspected, a judicious operator were entrusted with the case he might do much to prolong life and make it comfortable, even were it still too late to make a radical operation. If this be true of cancer of the stomach, it is equally true of most other visceral cancers in parts which are open to surgical attack.

Here, perhaps as well as anywhere else, fault might be found with one of the growing evils, as it appears to me, connected with the development of the last two decades. I refer to the Post Graduate Medical Schools, which are scattered through various parts of the country, where men, especially those to whom surgery appeals, either from one motive or another, spend a few weeks in witnessing abdominal operations, expecting then to go home and repeat the performances of the trained operators whom they for a brief time watched. I commend the acquisition of knowledge, but have had frequent reason to fear that it is some times followed by a determination on the part of the surgeon to "do", leaving it to the patient to "die." Suitable training for abdominal surgery cannot be obtained by a residence of a few weeks in New York City, or any other large capital.

Of course, you will say I have so far omitted all reference to that epoch-making operation of MacDowell's, who performed his first and historic ovariotomy 97 years ago, i. e., in 1809. So much has been said about it, and it is now so generally known and widely credited, that it seems hardly necessary to do more than mention it as perhaps the most historic event in surgery in America in the first part of the previous century. Of that to which it has led other writers of far more graphic power have written at length. It is to the everlasting credit of an American, as well as of America, and it is a great pity that Sydney Smith had not appreciated its significance ere he uttered his sneer. When in 1821 Nathan Smith did ovariotomy in Connecticut, while still ignorant of MacDowell's performances, upon which he improved by dropping the pedicle into the abdominal cavity, he certainly gave it great encouragement. Allan G. Smith also successfully imitated it in Kentucky, in 1823, and D. L. Rogers in New York, in 1829. Not until seven years later was the operation first performed in England, and not until fifteen years later in France. In the surgery of the ovaries and of the ovarian cysts much, nay nearly all, is due to the work of American surgeons. T. G. Thomas, in 1870, first devised and successfully made a vaginal ovariotomy, while Battey popularised vaginal oöphorectomy in 1872, and Sims contributed of his own methods and experience in making the vaginal route an effective and serviceable one for a variety of pelvic conditions.

Of further work within the pelvis it may be safely said that American surgeons have contributed more that is of value than those of any other nation. There is not an operation of any character which has not been greatly improved by them, while many of most distinctive value have been originated here. The greater part of this work has been done in New York by the surgeons of The Woman's Hospital, but much credit should be accorded, in this consideration, to the work and teachings of Price, Kelly, Martin, Dudley and many others.

While speaking of the pelvic organs we might as well, perhaps, say here that which can be said regarding the surgery of the prostate and of the bladder. In no country have the results of prostatectomy been more successful, nor the methods more simplified nor improved. To Belfield, to Goodfellow of San Francisco, to Alexander and Fuller of New York, and to many others, must be accorded the credit for either the introduction or the practical perfection of methods, both for suprapubic and perineal operations, which have made these radical operations comparatively safe and most satisfactory. If one may judge by the interest manifested at the meeting last year of The International Society of Surgery, in Brussels, there was no topic which men discussed more actively nor more interestedly than prostatectomy, while the three hundred surgeons present seemed equally divided in opinion as to the respective merits of the two routes. It is not proposed to go into this question here, but simply to record the fact that today there is scarcely a topic in surgery which evokes greater interest.

It was the work done by Otis, upon the normal calibre of the urethra, which led to the perfection by Bigelow of his method of litholapaxy, or evacuation by crushing in one sitting of vesical calculi. Until he introduced his large tubes for the purpose, this had not been practicable, not at least in a large proportion of cases. Bigelow's methods have not been improved upon within the past twenty-five years, thus constituting a notable exception to the work and the procedures of most surgeons. If one may refer to text-book work, the number of books published by American surgeons within the later decades, especially of the past century, is something more than memorable—it has become almost appalling. These works are not alone text-books for the student, but cover a large range—in fact, almost the entire range of surgical specialties, and bespeak an activity of study and an amount of work which has made it almost impossible for one man to be equally well versed in all directions. This has had much to do with the splitting up of general surgery into specialties which have, therefore, become almost a necessity. At the same time, the past ten years have witnessed one notable transition, i. e., the passing of one of these specialties. While gynecology is as much a study as ever, there are few men now who pose as specialists in this subject. They prefer to call themselves, rather, "Abdominal Surgeons," thereby illustrating the extent to which abdominal section has become a part of the treatment of the pelvic viscera, and indicating as well that the female pelvis is no longer regarded as a distinct cavity of the body into which no one might intrude, save he who posed as a gynecologist. As a matter of fact, this was originally a purely artificial distinction, having nothing to justify it save that the studies and tastes of certain great men took them solely in this direction, and that working in this field, they showed how it had been a neglected part of the body, and how general surgical methods and principles were equally applicable here as elsewhere. Thus, for a generation, gynecological surgery was made to appear as a distinct and sharply circumscribed kind of work and general operators were discouraged from attempting it. These artificial distinctions have been cleared away and it has been shown that the distinctly surgical part of gynecology is nothing more than is described above, whereas the non-surgical treatment of the diseases of women is becoming a rather neglected study, fascination attracting men to the former rather than to the latter line of work.

The development of the Medical Museum has done much to extend the capabilities of surgery. The museum is perhaps almost as old as the art, but the suitable collection and arrangement of specimens never became in itself an art until John Hunter taught not only how to correlate but to arrange specimens, in such a way that they should yield their full measure of instruction. The collections made in this country by Dr. Wood, in New York, and Dr. Waren, in Boston, rank among the largest individual enterprises of this kind and have each been the nucleus for museums of great importance; but the largest and the most creditable enterprise of this kind on this side of the ocean is due to the sagacity and industry of the officers of the Surgeon General's office in Washington, Drs. Woodward and Otis being mainly entitled to the credit for its beginnings.

What has been said of museums may be repeated also with regard to libraries. This country has now several large collections of medical books, which practically equal in value the most famous collections abroad. Our Army Medical Library in Washington is supposed to be superior to any in the world, while the collections contained in Philadelphia, New York, Boston and Chicago are most creditable and most valuable.

The most creditable publication in the world's medical literature belongs to this country; needless to say I refer to the Index Medicus,—both those colossal volumes which are now issuing in the second series from the Surgeon General's office, as a Government publication, and also that monthly publication which owes its inception to Billings and Fletcher, whose publication was for a short time suspended for lack of support, but which is now again resumed under the auspices of the Carnegie Institute. Surely no better use could be made of invested funds than the continuance of this great work, and surely none better appreciate its value than those who have occasion often to consult its pages. In this matter our profession and our Government lead the world.

We who are today so accustomed to calling in the services of a trained nurse, regarding it as the simplest and most natural of acts, are too likely to forget the tremendous aid which trained nursing has given to our art and the extent to which we now rely upon that which we, after all, have not so very long had within our grasp, i. e., the assistance afforded by trained nurses. Our State has had very much to do with the origin and development of that profession. The Bellevue school is generally credited with being the parent organisation in this country, it being the first in this country to grant a diploma. Its influence became widespread and most beneficial. There are now probably at least 20,000 graduate nurses in the United States, while the number of schools is constantly increasing. If nursing be included as a part of medicine then it may not be so necessary to make a division in the distribution of honors. However, it may be said that a considerable part of our advance during the last twenty-five years has been due to the improvement in general nursing which has been effected through the influence of these schools. The skilful trained nurse is the surgeon's best adjuvant, and until nursing can be dispensed with we shall never be able to dispense with her services.

One of the most important surgical developments of recent times has been the more rational treatment of cancer. While the etiology of this scourge of humanity is still not clear, and while men who are giving up their lives to its study are still disputing as to its nature and origin, we have nevertheless learned a great deal about it. It stands to the credit of the state of New York that it was the first government in the world to inaugurate an institution for the scientific study of cancer, and the State Laboratory for the Investigation of Cancer, in connection with the University of Buffalo, is entitled to the credit of being the pioneer institution of its kind in the world. As yet neither there nor elsewhere have been worked out such final results as to justify one in claiming positively any given origin for this disease. Without committing its staff, therefore, or without placing responsibility for the statement upon anyone else, I nevertheless feel no hesitation in saying that among the most careful workers and thinkers in the world today the parasitic theory is assuming an ever growing importance, and I say this advisedly, and in spite of the opposition which such a statement frequently creates, and in spite of objections which may be raised against it. The statement is the outcome of personal acquaintance or correspondence with the men whom I consider the keenest in this line of research, as well as of familiarity with what has been recently written by them and by others. The trouble has been that the profession are not yet ready for the statement, and one may easily prophesy that for a long time they will not receive it, not, at least, without most serious questioning, nor, for that matter, should they. Nevertheless all other lines of investigation lead up to mere negation, whereas work done in this direction leads ever to more and more fruitful results.

It is of sufficient importance to remind you here, by way of digression, that cancer occupies a unique position in the classification of diseases. It is perhaps the only disease of which it can be said that in and of itself it has no symtomatology; to which may be added that it has scarcely a distinctive sign by which it may with certainty be rcognised. One must take several factors into consideration in diagnosing even a superficial growth, and deep growths baffle the best diagnostic skill. In cancer of the stomach, for instance, there is not a single feature which is not met with in other cases, and the same is true of almost every organ of the body. This is a sad confession to have to make regarding a disease which kills annually in our own State six thousand individuals, but it lends plausibility to the general wisdom of making early explorations, since only in this way can the senses of sight and touch be brought into advantageous cooperation with the arguments obtained by inductive processes alone.

But it is not my object in this address to go deeply into this subject. It must be said, however, that the studies just alluded to have led to a very much clearer recognition of the clinical aspects of cancer, and, consequently, to much more accurate notions regarding its treatment. In some respects, at least, most surgeons are agreed—namely, that cancer is in the beginning a local disease, and, as I am fond of putting it, *if* it could be recognised in time, and *if* it could be made accessible, and *if* it could be early and thoroughly removed, it would be, in most cases, at least, a curable disease. However, the "ifs" in the above statement are exceedingly important, and the very nature of the disease makes it impossible to successfully apply these canons of treatment in many instances; but the statements do apply to all cancers which are within reach and which may be diagnosed early by any one of the various means or methods serviceable for the purpose. And matters have now gone so far in the direction of earlier recognition of, and surgical operations for deep seated cancers, especially within the abdomen and even the cranial cavity, that I have never hesitated to lay down the following rule, with a challenge to any one who may desire to controvert it—namely, that a well founded suspicion of deep seated cancer justifies, in the first place, an exploratory operation, directed, at first, toward its recognition, and, secondly, if possible, toward its releif. Were application more frequently made of this general principle by competent men (let us hope never by incompetent men) we should have fewer deaths from this awful disease.

The development of so-called military surgery, (under which name we will for the purpose include camp sanitation, military hygiene, and all that goes to make for the physical welfare of armies), has been another conspicuous feature of our growth. It has been impossible until very recently to introduce teaching of these subjects in our war schools at West Point, Annapolis and Leavenworth, and the lessons concerning the disastrous sacrifice of life caused by ignorance in this regard on the part of line officers, during the recent war with Spain, have not been utilised as they should have been. The Japanese have taught the world a lesson of the greatest value in these respects, and it remains now to be seen how quickly the world at large and, what is of most importance to us, our own government will profit by these lessons thus inculcated.

Another conspicuous feature of the century's growth and progress has been the development of the perfected modern hospital, which bears no more relation to the ancient institution of that name than does the modern microscope to the simple lens which was known to Roger Bacon. In Paris, at the beginning of the previous century, they had scarcely discontinued the practice of putting from four to six patients in one bed, as they used to do in the Hôtel Dieu, where it not infrequently happened that the convalescent and the dying lay side by side under one cover. The modern hospital is a complexity, evolved out of many particular features, and provides now for every want and every need that can be foreseen, for the isolation of the infected, the sterilisation of all apparel, and the protection of every patient from the possibility of harm from any other patient, while hospital construction calls now for the highest degree of architectural and constructural skill.

Hospitals provide mainly for local needs and are supported both by private and public generosity, but there come occasionally calamities and wars which make demands that no fixed hospitals can supply. It is to meet the need of such exigencies that the Red Cross Association was founded, and it has been in furtherance of its objects that devoted men and women have sacrificed their time, their labors, and even their lives, in doing good to others, relieving the distressed, caring for the sick and wounded, and demonstrating the noblest of human and humane traits. It is doubtful if altruism can reach a higher degree of development than has been evinced by those who have so nobly performed these duties.

But here this actually brief, though apparently long résumé of a century's progress must be brought to a close. I say "of a century's progress," when nearly all of the work has been done within the last third of it. The writer is painfully conscious that he has had to omit much, both in the matter of men's names as well as their deeds, which should find a place in such an epitome. He has found it far more difficult to be reasonably brief than to be fair and just to all. Much has, therefore, been omitted which we would gladly have included did space permit. The record as it stands is perhaps a jut one! at least the endeavor has been sincere to make it such.

So far we have dealt with the past and the present. What is to be said of the future?

Each Year Book of Surgery shows us that it is still too soon to speak, as did Erichsen, of Finality in Surgery. The records show that practically no part of the body which is accessible by any route has been omitted from experimental attack; time may still be required to indicate whether these attacks are to be frequently repeated or not.

The so-called "anatomical surgeon" of the past generation has practically found his limitations. Gross anatomy holds but little now in store for even the most conscientious student. Therefore, the day of the surgical anatomist and operator alone has passed. unless to his purely anatomical knowledge he has added much of collateral origin and interest. To every surgeon, however, more and more must come that final knowledge of anatomy gained only through the microscope, and employed to his advantage in studying, as well, the process of repair as of disease. Such additions, for instance, to our knowledge as have been afforded by recent investigations concerning the neurons have a far reaching significance which entitles them to the consideration of the surgeon as well as of the neurologist.

Into surgical pathology our surgeons must be led ever more and more deeply, sometimes by direct, again by devious ways. That the surgical laboratory will maintain a more and more important position, both in surgical teaching and in preparation for surgery, must go without saying. In surgery, as well as in general medicine, though probably in lesser degree, we shall learn that, to use a Hibernicism, the best way to treat disease is to prevent it. In so far as we draw nearer to a discovery of the foundations of life, the surgeon will profit with the biologist and the general practitioner. The future is too misty to say where we shall find ourselves in this regard at the end of the second century of our existence.

The tendency of late has been for the surgeon to drift away from the internist, for reasons which are obvious to all. He has, however, in my estimation, drifted too far and needs to come back into closer correlation with his colleague and closer comparison of methods with results. The pendulum has swung a little too far in the direction of divorce between medicine and surgery. There is work enough for each in his respective field, and it will be to the interests of humanity that they work rather toward than away from each other. Calm judgment, patience and discretion should go hand in hand with the most active research. Only by combination may the latter be made of its greatest possible avail.

Your orator is deeply regretful that he cannot be prophetic, but he regards it as unsafe to indicate what will be the trend of our thought or what changes will be made in our methods during the next hundred years. There is no field of work in which men are plodding more industriously and indefatigably than in the problems before us. Unfortunately, these problems are seldom simplified as they are one after another attacked, but seem to assume ever wider and far reaching proportions. Whether the relation of actual knowledge to inquiry and doubt will be materially altered one hundred years from now no one may safely say, but one may safely say, considering what a quarter of a century has afforded, that the completion of the present century will find our profession not merely endowed but actually burdened with facts of which today we have no glimpse. He may feel also that the practice of surgery has not been simplified, but sadly complicated and made more difficult. On one matter I am sure all will agree,—that is, that, foreseeing the complexities which are to still more thickly surround us, preparation for our work must be longer, more ardous in its beginnings, more comprehensive than that as yet provided by our schools,—in other words the lengthening and broadening of our medical curricula will be necessitated. In fact, the time is almost in sight when one must have attained years of not merely discretion but of ripened knowledge before he may feel that he is entitled to commence the practice of our art.

That New York State has contributed to the best of what there has been and is in this direction gives promise that she will not fail us in these respects in the future. It is fair, then, to hope that the Empire State may be wisely forehanded in this regard, since she has not been unmindful in the past of her duties in this direction. The destinies of our local profession have proven to have been in safe hands during the first one hundred years of our Society's existence. It is not asking nor expecting too much of those who follow us that our record will be maintained in this regard, and that New York State will prove herself ever in the van in recognising what is good, and maintain her standards in this and other matters pertaining to science.

Gentlemen, again I thank you deeply for the honor of addressing these words to you at this time, realising fully the impossibility of doing justice to such an occasion, but assurng you that I feel its significance to the fullest, even if I have shown my inability to rise to it.

510 Delaware Ave.

---

For Shock or Collapse.—Bartholow recommends atropine hypodermically, or the aromatic spirit of ammonia by inhalation or subcutaneously.—*Denver Medical Times.*

